# Characterization of Comorbid Posttraumatic Stress Disorder and Major Depressive Disorder Using Ketamine as an Experimental Medicine Probe ^†^

**DOI:** 10.20900/jpbs.20210012

**Published:** 2021-06-29

**Authors:** C. Sophia Albott, Sey Lee, Kathryn R. Cullen, Paul Thuras, Shmuel Lissek, Joseph Wels, Katrina J. Friedrich, Alyssa M. Krueger, Kelvin O. Lim

**Affiliations:** 1 Department of Psychiatry & Behavioral Sciences, University of Minnesota Medical School, Minneapolis, MN 55454, USA; 2 Minneapolis VA Health Care System, Minneapolis, MN 55417, USA; 3 Department of Psychology, University of Minnesota, Minneapolis, MN 55455, USA

**Keywords:** ketamine, stress disorders, stress biomarkers, trauma mechanism, post-traumatic stress disorders, treatment-resistant depressive disorder, magnetic resonance imaging, functional connectivity, neuroplasticity, pharmacologic actions

## Abstract

Comorbid posttraumatic stress disorder and major depressive disorder (PTSD + MDD) is the most common pathological response to trauma, yet despite their synergistic detriment to health, knowledge regarding the neurobiological mechanism underlying PTSD + MDD is extremely limited. This study proposes a novel model of PTSD + MDD that is built on biological systems shown to underlay PTSD + MDD and takes advantage of ketamine’s unique suitability to probe PTSD + MDD due to its rescue of stress-related neuroplasticity deficits. The central hypothesis is that changes in PTSD + MDD clinical symptoms are associated with functional connectivity changes and cognitive dysfunction and that ketamine infusions improve clinical symptoms by correction of functional connectivity changes and improvement in cognition. Participants with PTSD + MDD (*n* = 42) will be randomized to receive a series of six ketamine infusions or saline-placebo over three weeks. Pre/post-measures will include: (1) neuroimaging; (2) cognitive functioning task performance; and (3) PTSD, MDD, and rumination self-report measures. These measures will also be collected once in a trauma-exposed group including PTSD-only (*n* = 10), trauma-exposed-MDD (TE-MDD; *n* = 10), and healthy controls (HC, *n* = 21). Successful completion of the study will strongly support the concept of a biologically-based model of PTSD + MDD. The results will (1) identify functional imaging signatures of the mechanisms underpinning pathological responses to trauma, (2) shift focus from mono-diagnostic silos to unified biological and behavioral disease processes and, thus, (3) inform interventions to correct dysregulation of PTSD + MDD symptom clusters thereby supporting more precise treatments and better outcomes.

## INTRODUCTION

### Comorbid PTSD and MDD Is the Most Common Pathological Response to Trauma and Is Associated with Poor Clinical Outcomes

Comorbid posttraumatic stress disorder (PTSD) and major depressive disorder (MDD) represents a major public health burden as it is associated with poor clinical outcomes and substantial human disability. Lifetime PTSD rates in the general population range between 7% and 12% [1] while rates among combat exposed veterans are 19% to 30% [2]. Most individuals developing PTSD have comorbid psychiatric diagnoses. For example, among National Guard combat veterans diagnosed with PTSD, 85% meet criteria for at least one additional diagnosis with 70% meeting criteria for MDD [3]. Thus, the most common pathological response to trauma is comorbid PTSD and MDD (PTSD + MDD) [4–6]. PTSD + MDD is associated with greater clinical severity compared to either disorder alone [7,8]. Individuals with PTSD + MDD have increased suicide risk [9–11], worse functional impairment and lower global functioning [12–14], more severe symptoms of PTSD and depression [8,15–17], and increased healthcare utilization [18,19]. Further, the presence of either disorder diminishes treatment efficacy while increasing chronicity for the other [11,12,20,21].

Multiple theoretical models indicate PTSD + MDD is a coherent subtype of pathological responses to trauma. Whereas responses to trauma are heterogeneous [22,23], the high rates of PTSD + MDD comorbidity suggest an underlying shared deficit. Theoretical models explain the high rates of co-occurrence by additive symptom severity [13], associative learning dysfunction [24], and latent variable constructs [25]. The high rates of comorbidity that persist even after controlling for symptom overlap [26,27] suggest a primary neurobiological mechanism. There is debate regarding whether a primary neurobiological deficit predisposes one to develop PTSD + MDD [28] following trauma exposure or whether the cumulative effects of these disorders converge on a unitary mechanism [13]. The insufficient knowledge of this specific primary neurobiological mechanism underlying PTSD + MDD and how this mechanism may be modulated represents a knowledge gap. Without a clear understanding of this mechanism treatment outcomes for this common clinical presentation will remain poor.

### Poor Clinical Outcomes Associated with Comorbid PTSD and MDD Calls for a Better Understanding of Its Underlying Mechanism

**Figure 1.**
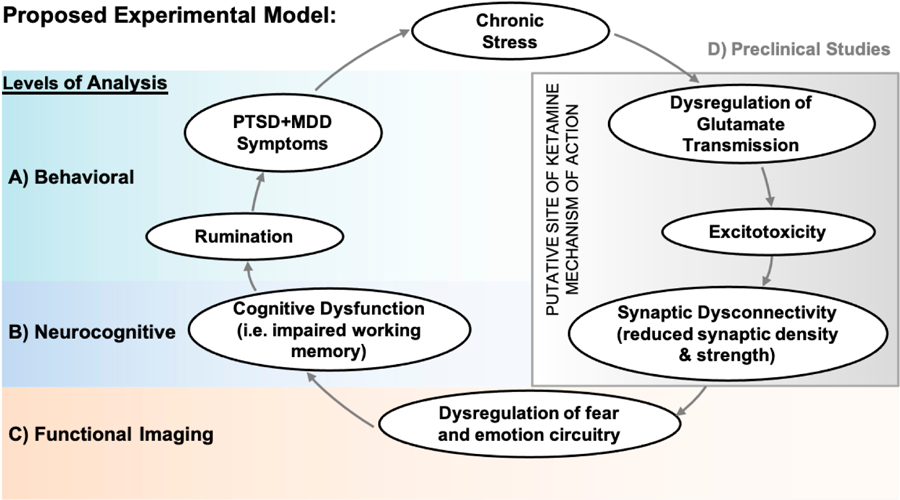
Schematic of this proposal’s experimental model assessing pathophysiological mechanisms underlying PTSD + MDD. Adapted from [29].

Our proposal suggests a “vicious cycle” model of PTSD + MDD that incorporates dysfunctional glutamatergic signaling, functional dysconnectivity, cognitive deficits, and behavioral symptoms to perpetuate a state of chronic stress (see Figure 1). This model unifies a diverse literature and highlights the biological mechanisms underlying this common but complex clinical presentation. Characterization of the processes described by this model will inform the development of interventions to correct the underlying pathophysiology thereby supporting more precise treatments and better outcomes.

### Conceptualization of PTSD + MDD

Preclinical models of chronic stress have implicated glutamatergic signaling dysfunction as key factors in the pathophysiology of PTSD and depression (see Figure 1D) [29–37]. This literature associates chronic stress with neuronal atrophy and decreased numbers of synapses in key corticolimbic brain circuits [38–40]. Neuronal atrophy and loss of glutamatergic synaptic connections catalyzed by stress have also been implicated in cognitive deficits associated with stress and depression [41]. Specifically, trauma-induced alterations of glutamatergic signaling combined with dysregulation of the hypothalamic-pituitary-adrenal axis are hypothesized to lead to decreased excitatory glutamatergic tone in the PFC (prefrontal cortex) [30,42] and hippocampus [30,37]. Moreover, hippocampal structural deficits in veterans with PTSD are associated with reduced global functional connectivity, PTSD and depression symptoms, and impaired memory [43,44].

Evidence suggests that PTSD + MDD is characterized by dysfunctional connectivity between the prefrontal cortex (PFC), hippocampus and amygdala (see Figure 1C). Neuroimaging research on PTSD + MDD has focused primarily on the corticolimbic structures of the amygdala, hippocampus, and PFC. The ventromedial PFC (vmPFC) and the amygdala are thought to form a functional network regulating emotional responses [45]. While the amygdala responds automatically to emotionally salient stimuli, the vmPFC inhibits this response in a context-dependent manner [45,46]. The vmPFC, thus, integrates internal and external representations of context, memory, emotion, and in so doing regulates behavioral and physiological responses [46,47]. In PTSD + MDD, reduced vmPFC activity reflects decreased control of amygdala responses to threat thereby resulting in a state of hypervigilance [45,46]. The dorsolateral PFC (dlPFC) exerts downstream control of the amygdala via effects on the vmPFC through cognitive emotion regulation.[48] Accordingly, the inability of the vmPFC to inhibit the amygdala in PTSD + MDD may be a down-stream effect of inefficient cognitive control by the dlPFC.

**Figure 2.**
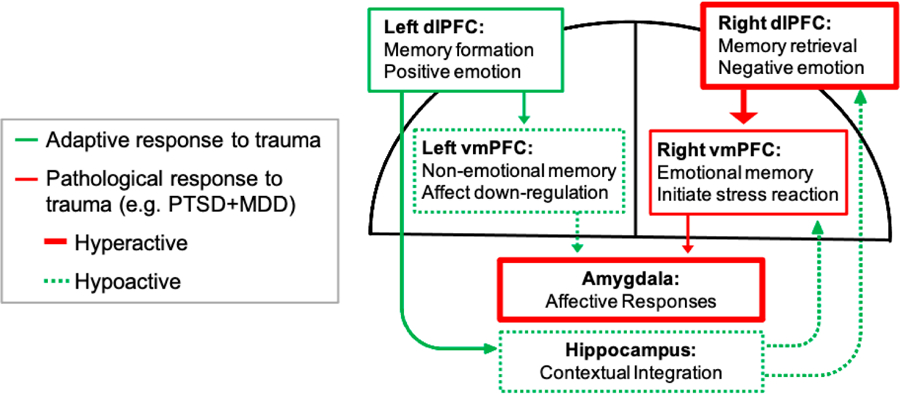
Schematic of the proposed lateralized functional dysconnectivity underlying PTSD + MDD (Adapted from [49]).

Converging evidence suggests lateralized dysfunction between the right and left dlPFC and the right and left vmPFC may occur in PTSD + MDD [49] (see Figure 2). A positron emission tomography (PET) study found that PTSD patients had decreased activation of left dlPFC compared to controls in a working memory task [50]. This finding was supported by evidence that the left dlPFC is affected by emotional distraction and may mediate impairments in cognitive function seen in PTSD + MDD (i.e., working memory updating) [51]. Furthermore, decreasing right dlPFC excitability with repetitive transcranial magnetic stimulation (rTMS) has been shown to be an effective therapy for depression [52] and to augment exposure therapy for PTSD [53]. The right vmPFC is implicated in processing emotional autobiographical memory, contextual integration, and control of autonomic nervous system responses to stress. Thus, elevated activity in the right vmPFC may render individuals more vulnerable to the negative effects of trauma, lead to impaired contextual integration of trauma memories, and undermine resilience. The hippocampus also plays a key role in the integration of contextual information during emotional memory encoding and retrieval. Aberrant connections between the left dlPFC, right vmPFC and hippocampus are thought to be involved with incomplete regulation of threat-related cues and heightened stress responsivity in individuals with PTSD+MDD. Taken together, it appears that frontal asymmetry in PTSD + MDD accounts for the inability of the PFC and hippocampus to effectively regulate the amygdala in a memory-guided and context-dependent manner.

### Functional Connectivity Deficits in PTSD + MDD Underlay Cognitive Deficits and Implicate Rumination as a Behavioral Mechanism Perpetuating PTSD + MDD Symptoms

Individuals with PTSD + MDD have greater neurocognitive impairment (e.g., verbal memory, attention) than individuals with PTSD or MDD alone [54–56] (see Figure 1B). It is proposed that neurocognitive deficits reflect downregulated functioning of the prefrontal cortex catalyzed by over activation of the amygdala. Cognitive resources in individuals with PTSD + MDD may be over-allocated to networks involved with emotional processing (i.e., the amygdala and prefrontal cortex) and under-allocated to cognitive control networks (i.e., the dorsolateral prefrontal cortex). This functional connectivity imbalance may lead to diminished neurocognitive integrity, specifically in information processing speed and working memory [57].

Consistent with the above-described alterations in functional connectivity, dysfunctional glutamatergic signaling has been associated with the cognitive deficits observed in PTSD + MDD [29]. Cognitive functioning deficits have generally been considered secondary sequelae of a primary psychiatric diagnosis. However, recent neurocognitive evidence suggests diminished cognitive control over information held in working memory may serve as a premorbid vulnerability factor in the development of these disorders [28]. Deficits in cognitive control provide a direct link to the proliferation of negative information (i.e., trauma memories, depressive cognitions) in working memory observed in rumination (the uncontrolled rehearsal of trauma-memories without extinction) which occurs in both PTSD and MDD. The centrality of cognitive control deficits to PTSD + MDD is supported by evidence that individuals with PTSD + MDD demonstrate greater neurocognitive performance impairment compared to individuals with either diagnosis alone [54–56].

Neuroanatomical and cognitive functioning deficits as observed in PTSD + MDD implicate rumination as a transdiagnostic vulnerability factor (see Figure 1A). Rumination is a maladaptive cognitive response style that maintains symptoms of depression [58–60] and predicts persistence of PTSD symptoms [61]. However, only two studies to date have examined the role rumination plays in PTSD + MDD. Roley et al. demonstrated that two rumination subtypes moderate the relationship between PTSD and MDD symptoms [62]. Birrer and Michael found heightened rumination in PTSD + MDD to also be a trigger for PTSD intrusion symptoms [63]. They concluded that rumination is a behavioral mechanism relevant for dysfunctional cognitive processes resulting from trauma and predisposing an individual to pathological reactions to trauma [63].

Evidence suggests dysfunctional connectivity in critical cognitive and emotional brain networks is related to maladaptive neuroplasticity insofar as it (maladaptive neuroplasticity) underlies a state of chronic stress associated with PTSD + MDD [64]. Preclinical models have shown that chronic stress alters neuroplasticity with reduced synaptic function in the hippocampus and PFC [62,65]. Clinical research also shows neuroplasticity dysfunction with altered functional connectivity (FC) in corticolimbic regions (i.e., PFC and hippocampus) associated with PTSD + MDD symptoms [43] as well as impaired learning (i.e., cognitive dysfunction) [4,5].

### The Case for Ketamine as a Probe to Characterize PTSD + MDD

The commonalities in biological systems characterizing PTSD + MDD support the use of similar classes of agents in therapeutics. Antidepressants, such as the selective serotonin reuptake inhibitors (SSRIs), are first-line pharmacological interventions for PTSD and MDD [66,67] but ineffective in a substantial proportion of individuals with either disorder [68,69]. Recognition of the inefficacy of standard antidepressant medications for MDD or PTSD has led to growing interest in the rapid antidepressant effects of ketamine—an *N*-methyl-D-aspartate (NMDA) type glutamate receptor antagonist [70–73].

Ketamine’s mechanism of action and clinical effects as a dissociative anesthetic and as an antidepressant occur at two different time scales. Acute administration of ketamine induces symptoms of dissociation, psychotomimetic experiences, and cognitive dysfunction. These acute effects are associated with direct administration of ketamine, hypothesized to be mediated via NMDA receptor antagonism, and have been shown to resolve rapidly (within 1 h) following cessation of ketamine [74]. However, ketamine administration has also been shown to trigger downstream signaling cascades that increase the expression of brain derived neurotrophic factor (BDNF) [75]. The expression of neurotrophic factors is thought to play a key role in maintaining optimal neuronal functioning including the protection and survival of neurons as well as for the induction of synaptic plasticity. In this sense, the acute dissociative effects of ketamine are *temporally dissociated* from the plasticity promoting effect of ketamine. This temporal dissociation is further reflected by the fact that ketamine’s peak antidepressant effect occurs 24 h after infusion cessation (Berman) with clinical effects persisting for 7 to 10 days following a single infusion [70,71,76].

Animal models of chronic stress demonstrate that ketamine rescues stress-related neuroplasticity deficits by increasing synaptic number and function in the rodent homologue of the prefrontal cortex [75,77–79]. Consistent with the animal model data, multiple mono-diagnostic studies have shown ketamine to be rapidly effective for ameliorating symptoms of MDD or PTSD [73,80]. Open label case series have also demonstrated that repeated (vs single) infusions are more clinically effective and enduring in individuals with treatment resistant depression (TRD) [81,82]. Moreoever, the reversal of stress-related plasticity deficits is reflected not only in amelioration of clinical symptoms but also in improvements in cognitive functioning, possibly reflecting restoration of synaptic function in key regions of networks subserving cognitive processing (such as the dlPFC). Thus, repeated ketamine infusions are uniquely suited to probe a model of PTSD + MDD.

*In support of the ketamine as probe characterizing our model of PTSD_MDD, we presented the first evidence that repeated ketamine infusions are effective for the rapid reduction of both PTSD and MDD symptoms in individuals with PTSD + MDD [83].* In this pivotal study, we demonstrated that six ketamine infusions in participants with PTSD + MDD results in significant improvement of both PTSD + MDD symptoms [83] and cognitive function [84]. Furthermore, our data suggested serial ketamine infusions to be safe and well tolerated; there were no worsening of dissociative, psychotomimetic, or mania symptoms during the treatment and follow-up periods. Despite classification of ketamine as a dissociative anesthetic agent, no worsening in dissociative symptoms was observed in any individual over the 8-week follow-up period.

Our secondary data analyses support the proposed model of PTSD + MDD, with covariation of PTSD and depression symptoms, cognitive dysfunction, and rumination. Our data (described below) provide face validity for a model relating functional dysconnectivity, rumination, and cognitive dysfunction with symptoms of PTSD and MDD. The model/conceptualization is further supported by the correction of these systems through repeated ketamine infusions. Our findings strongly dictate the need and utility of concurrently evaluating interrelated neurocircuitry, clinical phenomena (symptoms, rumination), and cognitive functioning using repeated ketamine infusions as a highly effective experimental medicine probe.

The foregoing background supports the scientific premise of a model connecting clinical symptoms observed in PTSD + MDD with cognitive dysfunction and altered functional connectivity as underpinning the dysfunction observed in this comorbidity. Our hypothesis—that clinical symptoms are associated with dysfunctional neurocircuitry and cognitive impairment in PTSD + MDD and that ketamine infusions improve clinical symptoms by correcting brain circuit dysfunction and poor cognition— provides a coherent model of a complex clinical presentation. The proposed research provides a strategy to characterize pathological responses to trauma and will permit determination of the validity of our hypotheses.

### Summary of Specific Aims

#### Specific Aim 1:

In PTSD + MDD, to examine how baseline clinical presentation, cognitive function and neurocircuitry predicts clinical response to ketamine infusions. We predict better clinical response (i.e., greater improvement of PTSD and depression symptoms as measured by the primary outcomes scales) will be associated with (**Hypothesis 1.1**) worse clinical symptoms (i.e., elevated total scores of rumination as measured by the Ruminative Response Scale, elevated total PTSD symptoms as measured by the Clinician Administered PTSD Scale for DSM-5, elevated total scores of depression as measured by the Mongomery-Asberg Depression Rating Scale), (**Hypothesis 1.2**) worse cognition and (**Hypothesis 1.3**) greater dysfunction in neural circuits.

#### Specific Aim 2:

In PTSD + MDD, examine the association of changes in corticolimbic circuitry with changes in clinical symptoms and cognition following either ketamine or saline infusions. We predict normalization of corticolimbic connectivity will be associated with (**Hypothesis 2.1**) improved clinical symptoms and (**Hypothesis 2.2**) improved cognition.

#### Specific Aim 3:

Examine cognition, rumination and neurocircuitry in trauma-exposed but diagnostically diverse groups (PTSD + MDD, PTSD-only, and trauma-exposed-MDD), and health controls. Compared with HC, PTSD + MDD will have (**Hypothesis 3.1**) impaired cognition, (**Hypothesis 3.2**) greater rumination and (**Hypothesis 3.3**) brain circuit dysfunction. An exploratory hypothesis is that TE-MDD and PTSD-only will be intermediate between PTSD + MDD and HC on these measures.

## INNOVATION

The innovation aspects of this project include: (1) method: the use of ketamine as an experimental medicine probe to characterize biological substrates underlying a coherent model of PTSD + MDD; (2) design: the application of pre- and post-treatment neuroimaging assessments to identify biomarkers predicting response to an empirically validated treatment for PTSD + MDD; and (3) concept: proposing a novel model of PTSD + MDD that is built on neuroanatomical and executive functioning systems implicated in the pathophysiology of PTSD + MDD. The proposed research is significant because it proposes a coherent model of PTSD + MDD that has the potential to advance our understanding of a homogeneous subgroup of individuals with pathological responses to trauma.

## APPROACH

### Preliminary Data: Improvement in PTSD + MDD Symptoms

**Figure 3.**
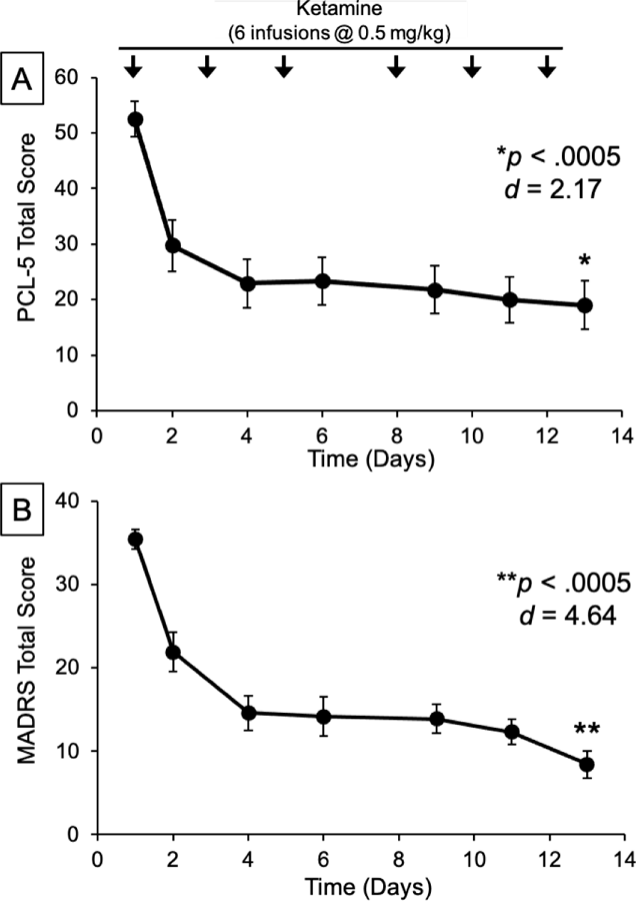
Changes in (A) PTSD symptoms as measured by the PCL-5 and (B) MDD symptoms as measured by the MADRS over the course of 6 ketamine infusions. Adapted from [83].

**Figure 4.**
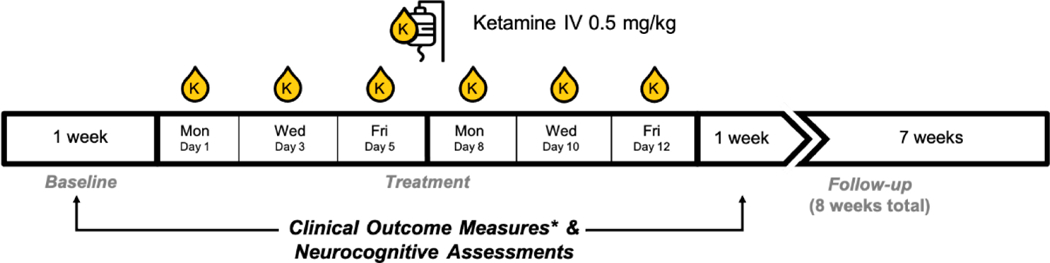
Schematic depicting pilot study design including timing of clinical and neurocognitive assessments in relation to the infusion series. Briefly, ketamine hydrochloride 0.5 mg/kg infusions were administered on a Monday, Wednesday, Friday schedule over two weeks. Clinical interviews (CAPS-5 and MADRS) and cognitive testing occured within 7 days prior to commencement of the infusion series and within 7 days following completion of the infusion series. Clinical measures of PTSD symptoms (using the PCL-5) and depression symptoms (MADRS) were also collected 24 h after each infusion.

An open-label sample of 15 veterans with PTSD + MDD receiving 6 ketamine infusions showed significant improvement in PTSD and depression symptoms (Figure 3). Pilot study design is depicted in Figure 4. Infusions occurred on days 1, 3, 5, 8, 10, and 12. Outcome measures (Montgomery-Åsberg Depression Rating Scale (MADRS) and PTSD Checklist for DSM-5 (PCL-5)) were collected 24-hours post-infusion, when the peak changes in clinical symptoms were hypothesized to occur. Cognitive testing occurred within 7 days before the infusion series began and was repeated within 7 days of infusion series completion. Participants were not washed out from psychiatric medications but, instead, required to be on stable doses for the 6 weeks preceding study entry and for the duration of study participation.

The mean within-subject change in PTSD symptoms significantly decreased from baseline to 24-h post-6th-infusion (Mean change in PCL-5 = 33.3, *p* < 0.0005, Cohen’s *d* = 2.17; see Figure 3A). Similarly, the mean within-subject change in depression symptoms significantly decreased over the infusion series (Mean change in MADRS = 26.6, *p* < 0.0005, Cohen’s *d* = 4.64; see Figure 3B).

**Figure 5.**
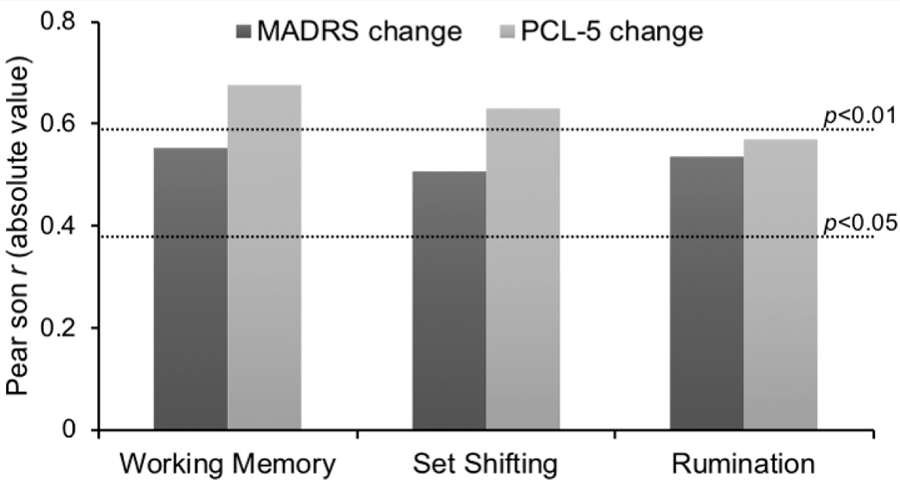
Correlations between baseline neurocognitive deficits (working memory, set shifting) and elevated rumination with change in PTSD and MDD symptoms after six ketamine infusions.

Baseline working memory deficits, worse set shifting, and increased rumination demonstrated significant correlation with improvement in PTSD and MDD symptoms after completion of the ketamine infusion series. Cognitive assessments occurred within 7 days of infusion series commencement and within 7 days of the final ketamine infusion. The mean time between baseline cognitive testing and commencement of infusion series was 5.1 days and the mean time between infusion series conclusion and post-treatment cognitive testing was 4.1 days. Individuals with greater deficits in a task for working memory and a task for set shifting were correlated with greater improvements in PTSD and MDD symptoms (MADRS change and working memory, *r* = −0.552, *p* < 0.05; PCL-5 change and working memory, *r* = −0.676, *p* < 0.01; MADRS change and set shifting, *r* = −0.506, *p* < 0.05; PCL-5 and set shifting, *r* = −0.63, *p* < 0.01). High levels of baseline rumination were also significantly correlated with greater improvements in PTSD and MDD symptoms (MADRS change and rumination, *r* = 0.535, *p* < 0.05; PCL-5 change and rumination, *r* = 0.57, *p* < 0.05) (see Figure 5).

## DESIGN

### Overview

**Figure 6.**
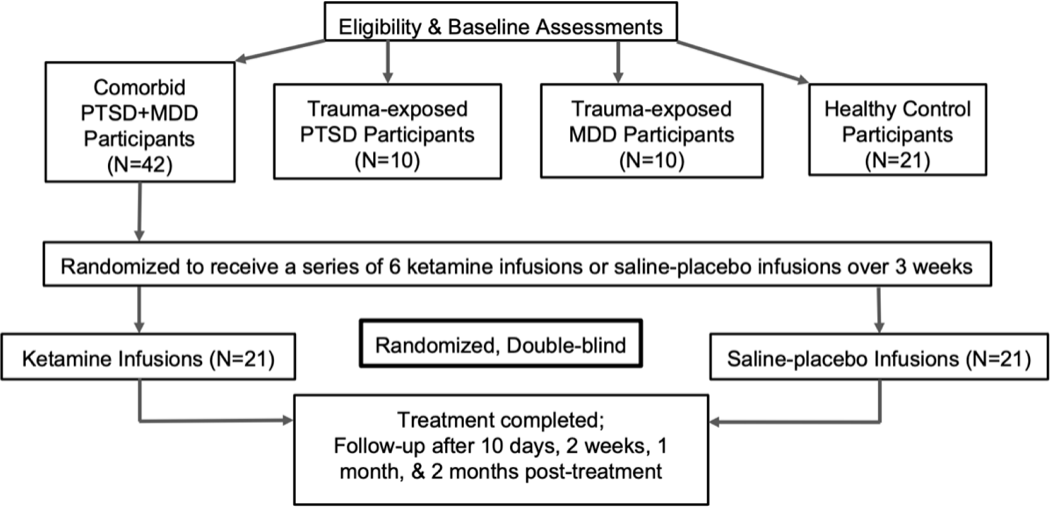
Overview of the study design.

This study is a double-blind randomized controlled trial (RCT) designed to identify neurocognitive and associated functional connectivity mechanisms underpinning PTSD + MDD outcomes to serial ketamine infusions. A total of 83 participants will be enrolled. PTSD + MDD participants (*N* = 42) will be randomized to receive 6 repeated infusions of ketamine or placebo over 3 weeks and be followed for 2 months. Healthy Controls (*N* = 21) will undergo identical evaluation and procedures except for infusion sequence and follow up. The same neurocognitive and functional connectivity mechanisms in a larger cohort of trauma exposed individuals will be examined with either MDD-only (*N* = 10) or PTSD-only (*N* = 10) participants. The experimental design is depicted in Figure 6.

### Participants, Screening and Baseline Assessment

Male or female veterans between the ages of 18 to 75 years will be recruited at the Minneapolis Veterans Affairs Medical Center (MVAMC) for this study. PTSD + MDD participants must meet Diagnostic and Statistical Manual of Mental Disorders, 5th Edition (DSM-5) criteria for MDD, single or recurrent, without psychotic features and DSM-5 criteria for chronic PTSD. The healthy control cohort will be recruited from the NIH ResearchMatch registry, flyers, and brochures. The trauma-exposed MDD-only participants must meet DSM-5 criteria for MDD, single or recurrent, without psychotic features and have experienced a traumatic event of sufficient severity to meet criterion A for a diagnosis of PTSD. The PTSD-only participants must meet DSM-5 criteria for PTSD.

To ensure eligibility, the Clinician Administered PTSD Scale for DSM-5 (CAPS-5) will be used to establish PTSD diagnosis. The Mini-International Neuropsychiatric Interview (MINI) will be used to confirm diagnosis of MDD and exclusionary diagnoses (e.g., current or lifetime diagnosis of psychosis-related disorder, bipolar I or II disorder, substance-induced mood disorder, or any mood disorder due to general medical condition). Cognition will be documented with a Mini-mental Status Exam (MMSE) score ≥ 27. Evaluation of exclusionary criteria involving unstable medical illnesses will be made based on medical record review by the principal investigator. Participants will be required to be on stable doses of psychiatric medications or to follow a consistent schedule of psychotherapy for 6 weeks preceding study entry and for the duration of participation. Other exclusionary criteria consists of (1) inability or unwillingness to provide written informed consent; (2) moderate/severe cognitive impairment; (3) history of moderate to severe traumatic brain injury, or other central nervous system (CNS) related disorder(s); (4) history of comorbid substance disorder within 1 month of screening; (5) prior use of ketamine as an antidepressant; (6) for participants of childbearing potential: pregnancy (confirmed by baseline lab test), or inability or unwillingness to use a medically accepted contraceptive method for the duration of the study; (7) imminent risk of suicidal/homicidal ideation and/or behavior; and (8) inability to undergo magnetic resonance imaging (MRI) (i.e., claustrophobia, ferromagnetic implants, etc.). Non-prescription drug use and pregnancy will be determined using urine screens.

Once eligibility is confirmed, primary outcome measures will assess depression and PTSD symptoms with the MADRS and PTSD Symptom Scale-Interview for DSM-5 (PSS-I-5), respectively. Secondary measures will include the Ruminative Response Scale (RRS), Inventory of Depression and Anxiety Symptoms (IDAS), Numeric Response Scale (NRS), Columbia Suicide Severity Rating Scale (C-SSRS), clinical impression of illness severity and improvement (CGI), PTSD Checklist for DSM-5 (PCL-5), Quick Inventory of Depressive Symptomatology Self Report (QIDS-SR16), Patient Reported Outcomes Measurement Information System-Sleep Disturbance Short Form (PROMIS), International Physical Activity Questionnaire-Short Form (IPAQ-Short), and General Anxiety Disorder-7 (GAD-7). Evaluation of cognitive function will occur via a battery of cognitive assessments using the NIH Examiner. Subtests include measures of multiple domains such as verbal memory, working memory, visual attention, task switching, reaction time, and risk taking; this battery was developed for repeat testing with extensive results concerning practice effects.

Neuroimaging will be completed using a 32-channel head coil on a 3T Siemens Prisma scanner at the Center for Magnetic Resonance Research (CMRR) at the University of Minnesota. All routine MRI safety screening procedures will be followed in their entirety to ensure the safety of participants. fMRI data acquisition will utilize multiband imaging, a technique that allows for dramatic increases in both spatial and temporal resolution of data acquisition. Multi-banded radiofrequency pulses can be used to accelerate volume coverage along the slice direction by simultaneously exciting and acquiring multiple slices and subsequently un-aliasing them using parallel imaging principles and the spatial information available in multi-channel radiofrequency (RF) array coils. This allows for a direct reduction in the volume repetition time (TR) by the number of simultaneously excited slices (i.e., the multiband (MB) factor or the slice acceleration factor). Encoding two or more images in each echo-planar imaging (EPI) echo train becomes highly efficient to reduce total scan time. The fMRI sequences will use MB factor of 8, allowing us to collect 32 slices in 1/8 TR that is 8 simultaneous slices in one TR. No special processing is required prior to analysis. These fMRI MB parameters will improve spatial resolution (from 3.5 × 3.5 × 4 mm^3^, 34 slices to 2 × 2 × 2 mm^3^, 72 slices with TR 270 MB = 8) and temporal resolution (from TR = 2000 to TR = 720 ms). Routine acquisitions on the Prisma scanner will also include collection of diffusion weighted imaging data using MB factor of 3. Diffusion weighted imaging (DWI) with MB may also improve spatial resolution with similar scanning time (Standard 30 dir, 6 B0, TR = 9000, 64 slices, 2 × 2 × 2 mm^3^ 6 min, New 1.5 × 1.5 × 1.5 mm^3^, 90 slices, MB = 3, 128 directions 16 B0, *b* = 1500, 8 min, TR = 3200). Reconstructed images from the Prisma scanner will be ready for standard processing. Participants enrolled in PTSD-only, TE-MDD, and HC groups will only undergo baseline assessments, including a single fMRI. A repeat fMRI will be completed in PTSD + MDD participants following completion of the intervention infusion series. See Table 1 for the full assessment schedule.

### Intervention Phase Assessment

During the treatment phase, PTSD + MDD veterans will arrive in the morning after an overnight fast. An indwelling catheter will be placed in their non-dominant arm for medication administration. The MVAMC Investigational Pharmacy will facilitate double-blinded randomization of the intervention for participants enrolled in the PTSD + MDD group. Measures of PTSD (PTSD Symptom Scale-Interview for DSM-5; PSS-I-5), depression (Montgomery-Åsberg Depression Rating Scale; MADRS) [85], pain (Numeric pain Rating Scale; MRS) suicidal ideation (Columbia Suicide Severity Rating Scale; CSSRS) and side effects will occur prior to the infusion and at 24-hours after each infusion. Participants will receive an IV infusion of either 0.5 mg/kg of ketamine hydrochloride solution or saline over 40 minutes. Potential side effects related to ketamine will be measured throughout the duration of the infusion using the positive symptom subscale of the Brief Psychiatric Rating Scale (BPRS+) [86], the Clinician-Administered Dissociative States Scale (CADSS) [87], and Young Mania Rating Scale (YMRS) [88]. To ensure safety, research staff will constantly monitor vital signs and the occurrence of side effects. Emergency medications and a crash cart will be available to manage unanticipated side effects. The infusion will be discontinued if adverse events do not respond to interventions. All participants will be monitored at least 2 hours post-infusion. Before leaving the infusion unit, participants will be required to demonstrate that all clinically significant side effects have resolved using the modified Aldrete scale (mAldrete) [89]. Post-infusion day measures will be obtained by telephone to reduce participant burden. These measures are administered according to the schedule described in Table 2. Following completion of the six infusion sequence, participants will attend post-treatment assessments for up to 2 months. Follow-up assessments are shown in Table 1.

### Statistical Analysis

#### Power Analysis.

The power analysis for Aims 1 and 2 was performed for PCL-5 and MADRS score using Nquery Advisor 4 (Statistical Solutions, 2000) under the following assumptions: (1) repeated measures ANOVA with the main effects of treatment (six infusions) and time (0 and Day 12), and the treatment by time interaction; (2) compound symmetric covariance matrix, and; (3) 5% significance level. In our pilot study, we observed a mean PCL-5 change of 33.3 (SD = 18.5) and a mean MADRS change of 26.6 (SD = 6.5) in a ketamine infusion group. Assuming the same effect size and variability in the ketamine group, we could detect a statistically significant effect when compared to an 8.8 point (or smaller) MADRS change or 11.7 point (or smaller) PCL-5 change in the placebo group. The estimates for the mean, standard deviation and intra-subject correlation obtained from a sample size of 21 patients per group will be required to detect a 10-point difference between the two groups in “change in MADRS score or change in PSS-I-5 from baseline to post-infusion” with 80% power to show a significant effect (α = 0.0125 Bonferroni corrected for 4 pairwise comparisons).

Planned statistical analyses for each Specific Aim are described below:

##### Specific Aim 1:

To examine how baseline clinical presentation, cognitive function and functional connectivity correlates with clinical response to ketamine infusions we will conduct Pearson correlations using PSSI total change scores, MADRS total scores, measures of different neurocognitive domains, and functional connectivity measures.

#### Cortico-limbic Functional Connectivity Analyses.

A seed-based approach will be used to examine functional connectivity within fronto-limbic neural networks [90]. Spherical seeds will be created for the dlPFC, vmPFC, the amygdala, and the hippocampus. Mean time series of each seed will be calculated by averaging across all voxels within the seed. Nine nuisance covariates (time series for global signal intensity, white matter, cerebrospinal fluid, and six motion parameters) will be included in our regression analyses to minimize the contributions of artifactual physiological signals (e.g., cardiac and respiratory cycles). To measure functional connectivity for each ROI, correlations will be determined for all other voxels in the brain, yielding a network of brain areas with highly correlated fluctuations in spontaneous BOLD signal. Connectivity maps will be compared between groups (ketamine-responders and ketamine-nonresponders). Group-level analyses will be carried out using a mixedeffects model as implemented in the FSL program FLAME. Age, gender, and concurrent psychiatric medication class will be included as statistical covariates in the analysis to ensure that any observed group effects are independent of age-related changes, gender, or concurrent psychiatric medication status. Corrections for multiple comparisons will be carried out at the cluster level for the networks correlated with each seed ROI using Gaussian random field theory (min *Z* > 2.3; cluster significance: *p* < 0.05, corrected).

##### Specific Aim 2:

To examine the association of changes in corticolimbic functional connectivity with changes in clinical symptoms and cognition following either ketamine or saline infusions, we will use linear mixed models to test for group differences in change over time of psychiatric symptoms, neurocognitive assessments, and neuroimaging (functional connectivity data) from baseline to post-infusion series. We will also carry the healthy control data forward over time to model the comparison between change in each group and a constant control condition. The analysis for each outcome will consist of maximum likelihood growth curve models that include group, time, and a group × time interaction as fixed effects, and the intercept and slope as random effects with an unstructured covariance matrix. The focus of these analyses will be to compare biomarker assessments/behavioral outcome measures between individuals that received the ketamine infusion series to individuals who received placebo. Post-hoc analyses will also examine covariates such as age, gender, and concurrent psychiatric medication class to ensure that any observed changes are independent of these factors.

##### Specific Aim 3:

To examine the relationship between clinical symptoms, cognition, rumination and neurocircuitry in PTSD + MDD, PTSD, TE-MDD, and HC at baseline, we will use multivariate analysis of covariance (MANCOVA) to test for group differences in transdiagnostic psychiatric symptoms (IDAS), neurocognitive assessments, and neuroimaging between participants and healthy controls. We will also run hypothesis-driven post-hoc tests for group differences on each measure.

## CONCLUSION: IMPACT AND FUTURE DIRECTIONS

Upon successful completion of this research study, we expect to establish a model of PTSD + MDD based on neurocognitive, neuroimaging, and behavioral measures that will characterize the pathology at baseline in a heterogeneous group of trauma-exposed individuals and following treatment response to ketamine in a more narrow group of participants with PTSD + MDD. Knowledge gained from this project will (1) identify functional imaging signatures of the mechanisms underpinning pathological responses to trauma, (2) shift focus from mono-diagnostic silos to unified biological and behavioral disease processes and, thus, (3) inform interventions to correct dysregulation of PTSD + MDD symptom clusters thereby supporting more precise treatments and better outcomes. Furthermore, these findings would set the stage for: (1) a larger scale study that could test whether the identified mechanisms applied to a more diagnostically heterogeneous sample of individuals with posttraumatic pathological responses supported by the data collected for exploratory Aim 3; (2) use of the identified neurocircuits/neurocognitive deficits as targets for novel treatment interventions; (3) use of objective pathophysiological markers for enriching the traditionally descriptive definitions of PTSD and depression; and (4) development of relatively inexpensive behavioral and cognitive biomarkers that could be applied in clinical settings. Indeed, these findings can be expected to contribute to the overall enhancement of clinical care by improving diagnosis and prognosis as well as positively influencing the discovery of novel treatment targets, thereby significantly reducing the burden of comorbid posttraumatic stress disorder and major depressive disorder.

## Figures and Tables

**Table 1.** Assessment schedule.

Construct	Measure	Eligibility and Baseline	Treatment Phase	Follow-Up Phase
		1–2 weeks before start of treatment	Day 1 to 13	Monthly × 2 months
**Primary Outcome Measures**				
Depressive Symptoms	MADRS		×	×
PTSD Symptoms	PSS-I-5		×	×
**Interview Based Assessments**				
Decision-making capacity	Modified Dysken Screening Tool	×		
MDD Diagnosis	SCID-CT	×		
PTSD diagnosis	CAPS-5	×		×
Rule-out moderate/severe cognitive impairment	MMSE	×		
**Side Effects and Secondary Outcome Measures**				
Side Effects (dissociative, psychotogenic, and manic symptoms)	CADDS, BPRS+, YMRS		×	
Internalizing symptoms	IDAS	×		×
Pain Intensity	NRS	×	×	×
Recovery from ketamine	mAldrete		×	
Suicide Risk	C-SSRS	×	×	×
Cognitive function	CogState	×		×
Rumination	RRS	×		×
Neuroimaging*		×		×*

* Within 1 week after completion of infusion series.

**Table 2.** Daily assessment schedule during infusion phase.

	Measure	*t*_0_-60 min	*t*_0_ + 40 min	*t*_0_+ 100 min	*t*_0_ + 160 min	*t*_0_ + 24 h
**Primary Outcome Measure**						
Depressive Symptoms	MADRS	×				×
PTSD Symptoms	PSS-I-5	×				×
**Secondary Outcome Measures**						
Pain intensity	NRS	×				×
Suicide risk	C-SSRS	×				×
**Hemodynamic Measures**						
BP, pulse, RR, SatO_2_		×	×	×	×	
**Side Effects Measures**						
Dissociative symptoms	CADSS	×	×	×	×	
Psychotomimetic symptoms	BPRS+	×	×	×	×	
Manic symptoms	YMRS	×	×	×	×	
Recovery from ketamine	mAldrete				×	

*t*_0_ = Infusion start.

## ABBREVIATIONS

PTSD: post-traumatic stress disorder
MDD: major depressive disorder
PTSD + MDD: comorbid post-traumatic stress disorder and major depressive disorder
TE-MDD: trauma-exposed major depressive disorder
HC: healthy control
PFC: prefrontal cortex
vmPFC: ventromedial prefrontal cortex
dlPFC: dorsolateral prefrontal cortex
rTMS: repetitive transcranial magnetic stimulation
TRD: treatment resistant depression
FC: functional connectivity
MADRS: Montgomery-Åsberg Depression Rating Scale
PCL-5: PTSD Checklist for DSM-5
MVAMC: Minneapolis Veterans Affairs Medical Center
DSM-5: Diagnostic and Statistical Manual of Mental Disorders, 5th Edition
CAPS-5: Clinician Administered PTSD Scale for DSM-5
MINI: Mini-International Neuropsychiatric Interview
MMSE: Mini-Mental Status Exam
PSS-I-5: PTSD Symptom Scale-Interview for DSM-5
RRS: Ruminative Response Scale
IDAS: Inventory of Depression and Anxiety
NRS: Numeric pain Rating Scale
C-SSRS: Columbia-Suicide Severity Rating Scale
CGI: Clinical Global Impression Scale
QIDS-SR16: Quick Inventory of Depressive Symptomatology Self Report
PROMIS: Patient Reported Outcomes Measurement Information System—Sleep
IPAQ-short: International Physical Activity Questionnaire-Short Form
GAD-7: Generalized Anxiety Disorder-7
CMRR: Center for Magnetic Resonance Research
RF: radiofrequency
TR: repetition time
MB: multiband
EPI: echo-planar imaging
DWI: Diffusion Weighted Imaging
BPRS+: positive symptom subscale of the Brief Psychiatric Rating Scale
CADSS: Clinician-Administered Dissociative States Scale
YMRS: Young Mania Rating Scale
mAldrete: modified Aldrete
MANCOVA: multivariate analysis of covariance
NIMH: National Institute of Mental Health
